# Habitual behavior of household food expenditure by store type in the United States

**DOI:** 10.1371/journal.pone.0291340

**Published:** 2023-09-08

**Authors:** Keehyun Lee, Oral Capps

**Affiliations:** 1 Department of Agricultural Economics, University of Kentucky, Lexington, KY, United States of America; 2 Department of Agricultural Economics, Texas A&M University, College Station, TX, United States of America; Southeast University, BANGLADESH

## Abstract

We examine how socio-demographic factors, spending habits, and characteristics of the retail food environment affect household expenditure across all food and beverage categories by store outlet in the United States. The six outlets considered are grocery stores, convenience stores, discount stores, club stores, drug stores, and dollar stores. The source of data for this analysis is the Nielsen Homescan Panel over the period between 2011 and 2015. We employ a dynamic correlated random effect Tobit model to incorporate habitual purchasing behavior as well as a novel method to deal with zero observations using the inverse hyperbolic sine transformation. The results suggest that habitual spending behavior undoubtedly is a key factor in affecting food and beverage expenditures across all store outlets. Additionally, household size, age, urbanization, education, race, ethnicity, and region are drivers of household food and beverage expenditures across the six store outlets.

## Introduction

**Fig 1:**
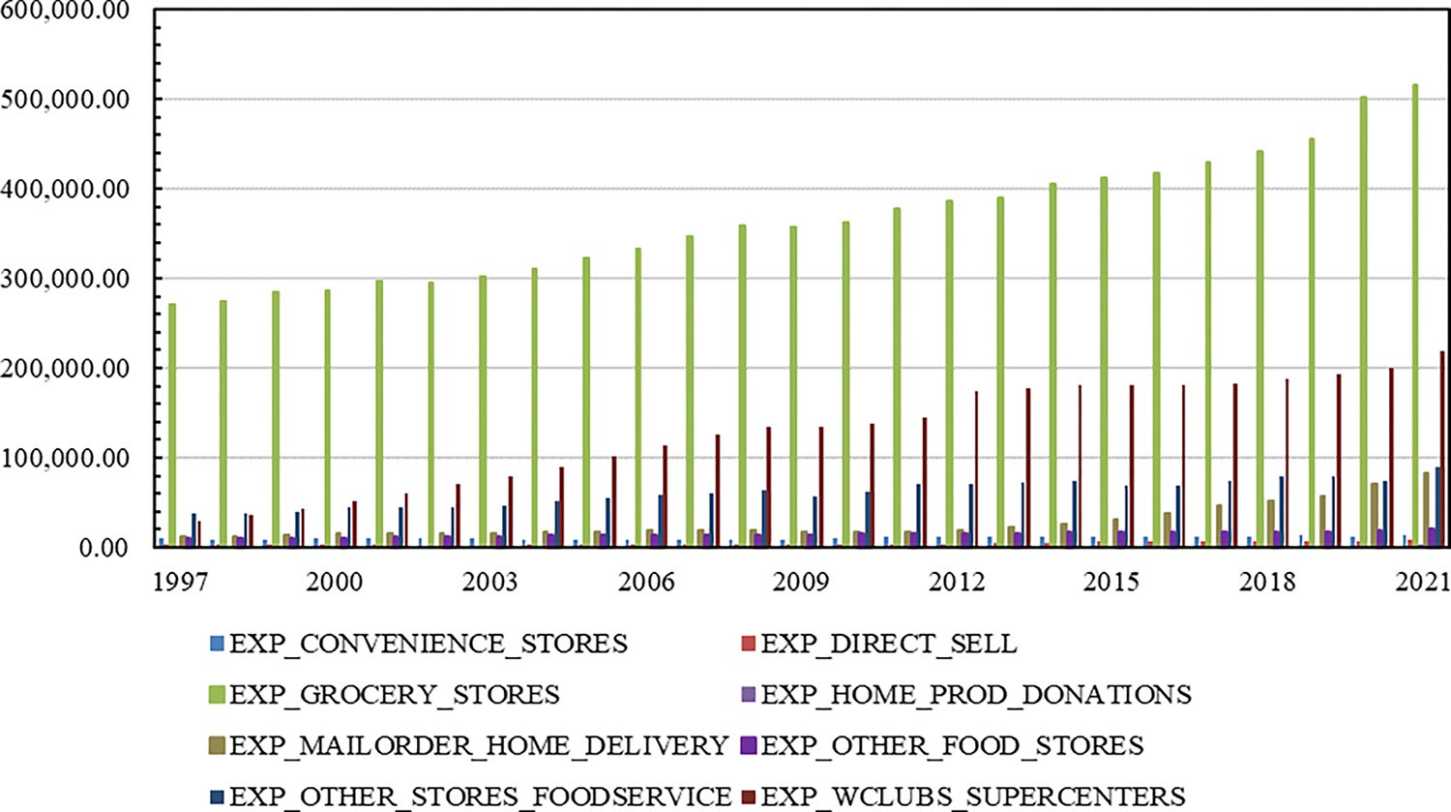
Breakdown of nominal food at home expenditures, 1997 to 2021 (millions of dollars). Source: Economic Research Service, United States Department of Agriculture. Okrent, A.M., H. Elitzak, T. Park, and S. Rehkamp, 2018. *Measuring the Value of the U*.*S*. *Food System*: *Revisions to the Food Expenditure Series*, Technical Bulletin (TB-1948), September, 72 pp.

Without question, the food retail environment has changed over the past few decades [1–3]. Over the past 25 years, several nontraditional store formats—including supercenters (such as Wal-Mart), dollar stores, and club stores—have gained market share and prominence in the retail food landscape. As exhibited in Fig 1, the Economic Research Service (ERS) breaks down nominal food expenditures into eight categories: (1) convenience stores; (2) grocery stores; (3) mail order/home delivery; (4) warehouse clubs/supercenters/mass merchandisers; (5) direct sales; (6) other food stores; (7) other stores foodservice; and (8) donations. Over the period 1997 to 2021, nominal expenditures from convenience stores were $10.86 billion on average; currently $14.67 billion; from grocery stores $365.14 billion on average; currently $515.43 billion; from mail order/home delivery $27.68 billion on average; currently $83.68 billion; from warehouse clubs/supercenters/mass merchandisers $129.52 billion on average; currently $219.85 billion.

**Fig 2:**
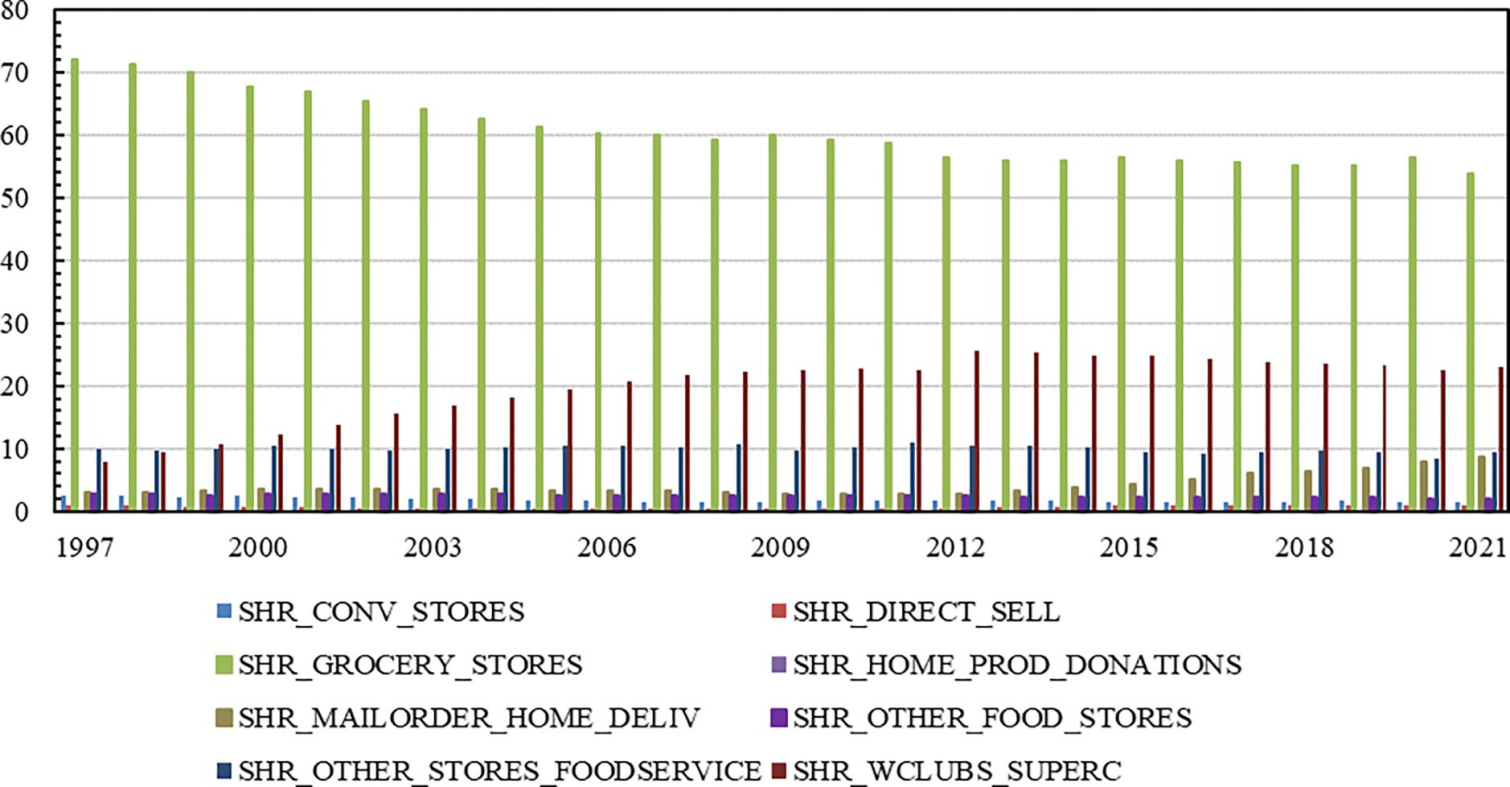
Share of nominal food at home expenditures, 1997 to 2021, percent. Source: Economic Research Service, United States Department of Agriculture. Okrent, A.M., H. Elitzak, T. Park, and S. Rehkamp, 2018. *Measuring the Value of the U*.*S*. *Food System*: *Revisions to the Food Expenditure Series*, Technical Bulletin (TB-1948), September, 72 pp.

As shown in Fig 2, shares of nominal food at home expenditures over the period 1997 to 2021 were as follows: (1) convenience stores, 1.84% on average, ranging from 1.44% to 2.59%; currently 1.54%; (2) grocery stores, 60.60% on average, ranging from 53.94% to 72.00%; currently 53.94%; (3) mail order/home delivery, 4.16% on average, ranging from 2.72% to 8.76%; currently 8.76%; and (4) warehouse clubs/supercenters/mass merchandisers, 19.92% on average, ranging from 7.99% to 25.50%; currently 23.00%. Accounting for about 77% of at-home food expenditures at present, the major outlets unequivocally are grocery stores and warehouse clubs/supercenters/mass merchandisers. But the share of nominal food expenditures from grocery stores has been on the decline over the period 1997 to 2021, while the share of nominal food expenditures from warehouse clubs/supercenters/mass merchandisers has been relatively constant since 2017. The share of nominal food expenditures from convenience stores also has been relatively constant since 2015. On the other hand, the share of nominal food expenditures from mail order/home delivery has experienced a notable rise from 3.20% in 2013 to 8.76% currently, as well, longstanding outlets such as convenience stores, discount stores, and dollar stores have expanded their food offerings to better attract grocery shoppers [3].

The sole purpose of this study is to examine how socio-demographic factors, spending habits, and characteristics of the retail food environment affect household expenditure across all food and beverage categories by store type. Whether traditional or nontraditional, store outlets differ in prices, product assortment, advertising strategies, and location [3]. The various outlets considered in this study are grocery, convenience, discount, club, drug, and dollar store types. The source of data for this analysis is the Nielsen Homescan Panel over the period between 2011 and 2015. Specifically, we form a balanced panel of 28,109 households who participated in the survey for all five years from 2011 to 2015. The total number of observations available for analysis is 140,545.

Previous studies from the fields of economics and marketing have mainly centered attention on the determinants of store choice. Evidence from this rich literature suggests in large part that the choice of food stores is based on a variety of factors including prices, product variety, quality of meat and produce, distance from home, courteous services, and degree of competition [4–12]. Store choice also has been shown to be influenced by household demographics and past purchase history [13] as well as by characteristics of the entire local food market [14–16], the degree of competition among food stores [7], and prices offered by various outlet types [17–20]. Additionally, previous studies have investigated the role that food access plays in food insecurity, malnutrition, and fruit and vegetable consumption, among other concerns [21–23].

Taylor and Villas-Boas [24] investigated choices of store outlets as a function of household attributes using a multinomial mixed logit model based on data acquired from the National Household Food Acquisition and Purchase Survey (FoodAPS). Household attributes included participation in the Supplemental Nutrition and Assistance Program (SNAP), household income at various levels of the Federal Poverty Line (FPL), and various measures of the food environment and food access—population density, the share of households living in rural and urban census tracts, the share of households living in a census block group identified as a food desert and share of households without car access. The store outlets considered were supermarkets, superstores, grocery stores, convenience stores, and farmers’ markets.

Moreover, based on data from a panel of 3,376 households collected from 11 randomly selected mid-sized counties in the United States, Fan [25] analyzed the effect of improving food accessibility by way of subsidizing purchases of fruits and vegetables across food deserts and non-food deserts. The household panel was compiled from 174 food stores collected using scanning devices from Information Resources, Inc. (IRI InfoScan) over a period of 16 quarters from 2009 to 2012 in the 11 sample counties. Data pertaining to store characteristics came from the Nielsen TDLinx store directory and census-tract level socio-demographic information was obtained from the 2008–2012 American Community Survey (ACS). Census-tract level food deserts indicators were compiled from the 2010 USDA Food Access Research Atlas (FARA). The principal conclusion was that expanding the availability of fruits and vegetables in the nearest stores of food deserts without changing prices did not affect appreciably store choice or enhance the welfare of the household panel. In contrast, price subsidy programs associated with fruits and vegetables in food deserts improved the welfare of household panelists.

Volpe, Kuhns, and Jaenicke [3] examined the effect of store format and income on the *healthfulness* of food purchased based on a large nationwide sample of households as recorded by the Information Resources, Inc. (IRI) over the period between 2008 and 2012. The healthfulness measures used were based on the Low-Cost, Moderate-Cost, and Liberal Food Plans 2007 [26] developed by the Center for Nutrition Policy and Promotion, USDA as well as the Healthy Eating Index developed by the USDA in 2005 [26]. Correlations between store formats and the respective healthfulness measures as well as correlations between store formats and expenditure shares by food category were presented. The store formats in this study were supermarkets, drug stores, mass merchandisers, supercenters, convenience stores, dollar stores, and club stores. Despite the wealth of descriptive information provided, Volpe, Kuhns, and Jaenicke [3] did not provide a formal econometric analysis.

Finally, Volpe, Jaenicke, and Chenarides [27] investigated the relationship between store formats and the healthfulness of at-home food purchases. The store formats used in this study were supermarkets, drug stores, mass merchandisers, supercenters, club stores, convenience stores, and other stores. To investigate the healthfulness of household food purchases, based on the methodology developed by Volpe and Okrent [28] a healthfulness score was assigned, hereafter called the USDA*Score*, to the shopping baskets of each household by quarter. The source of data for this analysis was the Nielsen Homescan Panel over the period between 2004 and 2010. The USDA*Score* is based on the differences between category-specific observed expenditure shares and USDA recommended expenditure shares. The goal was to investigate how store format decisions and other factors affect the household specific USDA*Score*. The principal conclusion drawn from this analysis was that healthier food choices were associated with higher food expenditure shares at supermarkets and supercenters and lower food expenditure shares at drug stores and convenience stores. In addition, increased retail food industry concentration had a negative effect on shopping healthfulness.

Despite these previous studies that largely focus on factors affecting store choice, one area of research that has received relatively little attention is how the ***magnitude of household food expenditures*** is impacted by store formats and store characteristics. To differentiate our study from the extant literature, we explore the factors which directly affect household food expenditure by store outlet. Indeed, Volpe, Jaenicke, and Chenarides [27] estimated the impacts of *expenditure share* by store format, but in our study, we quantify the magnitude of the impact of socio-demographics, the retail food environment, and spending habits on household food and beverage expenditures by diverse store types. Hence, by analyzing factors that impact household food expenditure across the aforementioned six store types, this study adds to the economic literature. Importantly, our study also considers habitual persistence or spending habits, a dynamic property of household food expenditures. However, in the previously mentioned studies, habitual behavior was not included in the set of explanatory variables.

To further differentiate our study from previous studies, we employ a dynamic correlated random effect Tobit model to consider habitual purchasing behavior. The panel structure allows us to incorporate dynamics by including lagged dependent variables as explanatory variables to account for spending habits. Another advantage of the use of this model is its ability to handle corner solution problems. The dependent variables, which reflect household purchasing history according to store type, contain both non-zero and zero values.

Initially, we provide definitions of the respective store outlets. Subsequently, we provide the theoretical framework and the empirical model for this study. Then we describe the Nielsen Homescan data, the construction of the balanced panel of households, and present descriptive statistics of model variables. Issues associated with the estimation of the dynamic correlated random effect Tobit model are discussed next. Following this discussion, the empirical results are presented. Finally, concluding remarks are made along with a discussion of study limitations and possibilities for further research.

## Materials and methods

Definition of store types

While there are no universally accepted definitions and classifications of food retail store formats, throughout this study we use the store format names provided by Nielsen, the vendor responsible for the collection of the Homescan data. A traditional supermarket is a food retailer with more than 9,000 square feet of selling space and at least $2 million in annual sales. Drug stores feature prescription-based pharmacies but generate at least 20 percent of their total sales from other categories, including general merchandise and food. Discount stores are mass merchandisers and typically large department stores (e.g., Target) that sell primarily general merchandise and nonperishables but also carry limited assortments of grocery products. Supercenters also have been known as hypermarkets and superstores are the largest formats, in terms of both square footage and product volume. Supercenters are hybrid stores that combine mass merchandisers with full supermarkets. These stores have a reputation among consumers for stressing low prices and convenience over consumer service [29].

Convenience stores are the smallest of the major retail formats in terms of size and product offerings and feature a limited selection of staple foods as well as ready-to-eat, prepared foods (e.g., hot dogs). Additionally, convenience stores sell general merchandise and, in many locations, alcohol, and tobacco. Dollar stores range in size and product variety, placing emphasis on low prices and offering little in the way of customer service. As the name suggests, many products in these stores cost one dollar. Club stores, also referred to as warehouse or volume stores, are large-format outlets that specialize in selling food and selected general merchandise. The grocery line features foods and beverages in bulk for relatively low prices. A feature of this format unique in food retailing is that memberships must be paid to shop there.

### Theoretical framework

Based on household production theory, the expenditure function for any commodity is the product of derived demand for factor inputs and the corresponding price vector of factor inputs [30–32]. Let the commodity in question be all food and beverages purchased by household *h* at store outlet *k*. Then as given by Eq (1), household expenditure at store *k* may be written as

$${EX}_{hk}=P_{hk}X_{hk}=g({P_{hk,}Y}_{h},W_{h},D_{h},E_{h})$$ (1)

where *X*_*i*_ is the derived demand of factor inputs for household *h* at store outlet k, *P*_*hk*_ is the price vector of factor inputs paid by household *h* at store outlet *k*, *W*_*h*_ is a measure of the opportunity cost of time of household *h*, *Y*_*i*_ represents the income level of household *h*, *D*_*h*_ represents the set of socio-demographic characteristics of household *h*, and *E*_*h*_ represents the retail environment faced by household *h*.

Household heterogeneity typically is accounted for incorporating socio-demographic variables in the theoretical model. Hill and Lynchehaun [33] identified various cultural and socio-economic factors influencing consumer preferences including age, ethnicity, income, education, gender, absence/presence of children, marital status, region, and race. In particular, education reflects knowledge about health and nutrition [3, 31, 32, 34, 35]. Similar to Volpe, Janeicke, and Chenarides [27], we include household income, household size, age, urbanization, race and ethnicity, region, and education in the set of socio-demographic variables in this study.

Additionally, in our theoretical model, we consider the potential importance of the retail environment in the household expenditure function. The retail environment represents the number of stores in the area in which the household lives; accessibility to store outlets may affect household production and consequently, household purchases of food and beverages. In this study, to address the impact of the retail environment, similar to Volpe, Jaenicke, and Chenarides [27], we count the number of supermarkets and other grocery stores, convenience stores, drug stores, and warehouse club stores based on zip codes.

Past studies related to the choice of store outlet did not account for habitual purchasing behavior [24, 25, 27]. Habits refer to repetitive behavior in purchasing and consumption behavior [36]. The habitual behavior of consumer purchasing patterns has been studied widely in the field of psychology [37, 38]. This repeated purchasing behavior has been investigated in a wide range of products and services [39–45].

To account for habitual purchasing behavior, we introduce a one-period lagged dependent variable in the model [46, 47]. As such, we augment the expenditure function given in Eq (1) for household h for store outlet k in time period t as:

$${EX}_{hkt}=h({EX}_{hk,t-1},P_{ht},Y_{ht},W_{ht},D_{ht},E_{ht})$$ (2)

*where EX*_*hk*,*t*−1_ is the one-period lagged expenditure variable for household h for each store type k in time period t.

### Empirical model

Given the focus of our research on analyzing the impacts of habitual spending behavior, the retail environment, and household heterogeneity by store outlets, we employ a dynamic correlated random effect Tobit model. This model specification allows us to deal with dynamics, panel data, and data censoring issues simultaneously accounting for household demographic variables and retail environment variables as explanatory variables. The model also accounts for potentially household-specific unobserved heterogeneity. As well, conventional fixed effect nonlinear models such as probit, logit, and Tobit models can produce biased estimates of structural parameters [48]. The use of the dynamic correlated random effect Tobit model circumvents this deficiency and produces consistent estimates of structural parameters.

Owing to the number and heterogeneity of purchases of specific food items and beverages as well as the censored observations associated with household food and beverage expenditures by store outlets, we omit prices from the model. In the Nielsen data prices are derived as the ratio of expenditures to quantities purchased. By omitting prices from the model, we avoid making imputations of missing prices and we avoid the potential endogeneity of prices with household expenditures. Simply, we assume that the impact of the price is implicitly captured by the type of store outlet.

One issue we encountered with our censored dependent variables is the irregularity of household expenditure in certain store types, specifically convenience stores and dollar stores. This irregularity contributes to a higher number of censored dependent variables. Consequently, the number of observations for the uncensored portion of the dependent variables becomes limited, posing challenges for the convergence of nonlinear estimation at times. We transform the dependent variables which include zero-valued observations using the inverse hyperbolic sine (arcsinh) mechanism [49].

A notable problem with taking the logarithm of any variable is that it does not allow retaining zero-valued observations because ln(0) is undefined. As pointed out by Bellemare and Wichman [49], “applied econometricians are typically loath to drop those observations for which the logarithm is undefined.” Consequently, researchers often have resorted to ad hoc means of accounting for this situation when taking the natural logarithm of a variable, such as adding 1 to the variable prior to its transformation [50]. In recent years, the inverse hyperbolic sine (or arcsinh) transformation has grown in popularity among applied econometricians due to the fact that it is similar to the behavior of the logarithm function, it allows retaining zero-valued observations without any arbitrariness, and it often results in normal distributions [49, 51–57].

**Fig 3:**
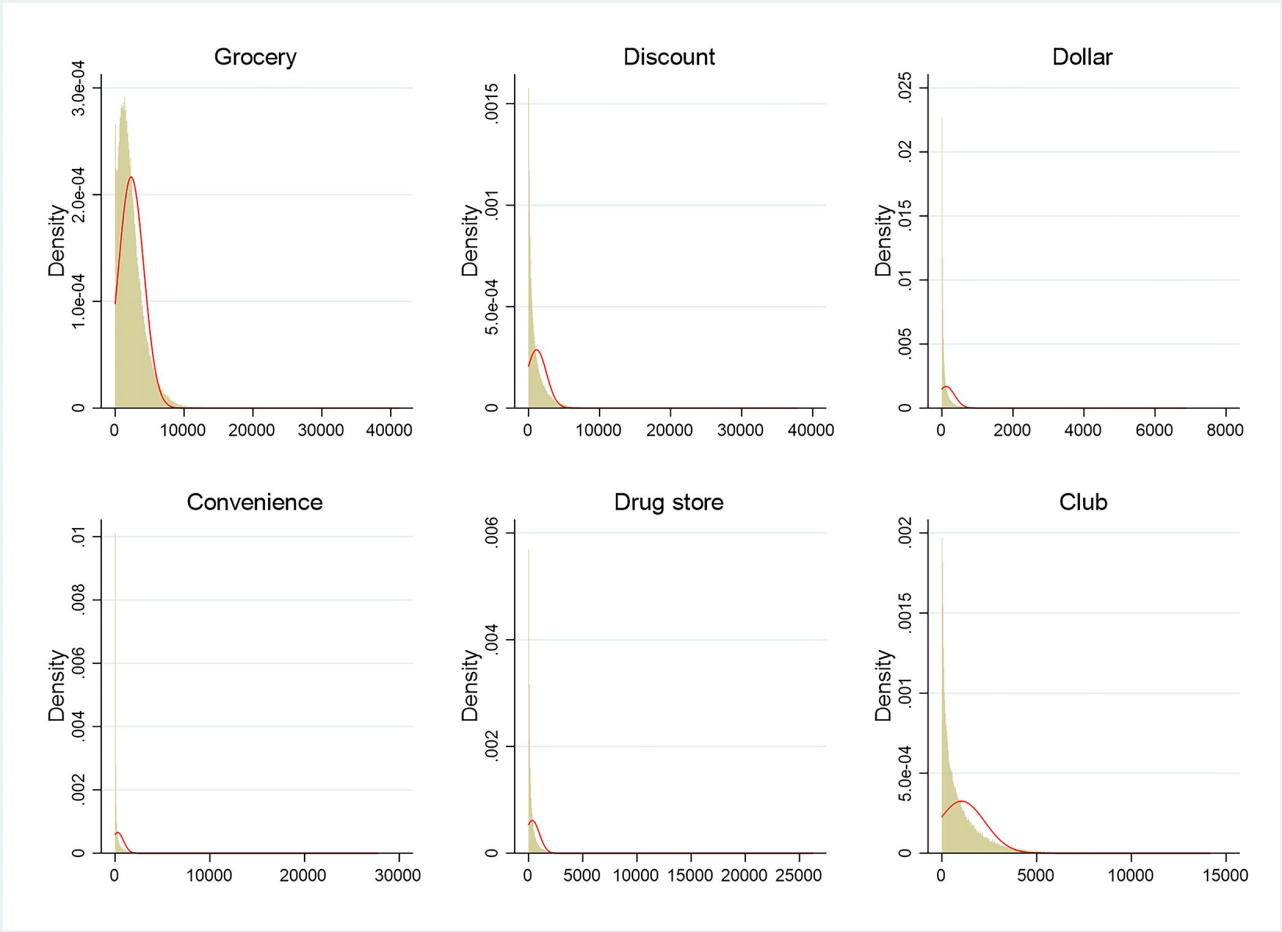
Distributions of household food expenditure associated with the six store types. The horizontal axis represents household expenditure.

**Fig 4:**
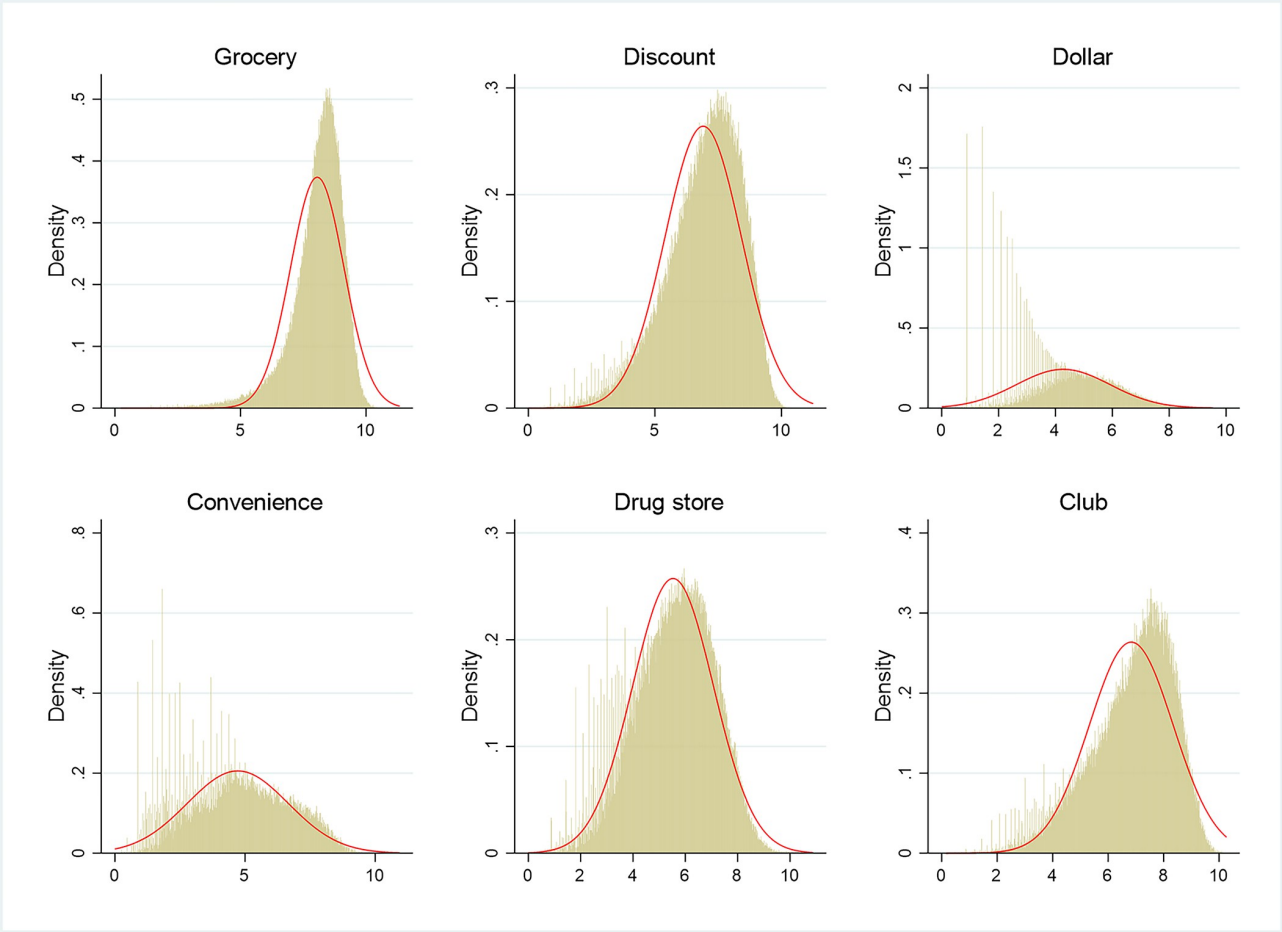
Distributions of transformed (inverse hyperbolic sine) household food expenditure associated with the six store types. The horizontal axis represents household expenditure.

Figs 3 and 4 show the distributions of the original and transformed household food expenditure associated with each store type conditional on expenditures above zero (the horizontal axis indicates expenditure). In Fig 3, only food expenditures from grocery stores follow a truncated normal distribution. In Fig 4, after implementing the inverse hyperbolic transformation, all six dependent variables appear to follow normal distributions. In addition, the zero values of the dependent variables still remain as zeros with the inverse hyperbolic transformations. So, by transforming our dependent variables pertaining to household food expenditures, we can deal with corner solution issues in our data using the Tobit model.

Using Eq (3), we express the transformed household food and beverage expenditure variables by store type based on the inverse hyperbolic sine method. We denote ${\bar{EX}}_{ht}^{k}$ as the dependent variables for household h, store outlet k, and time period t in our empirical model.


$${\bar{EX}}_{ht}^{k}=arcsinh({EX}_{ht}^{k})=ln({EX}_{ht}^{k}+\sqrt{{{EX}_{ht}^{k}}^{2}+1})$$ (3)

Initially, we start from the definition of our latent variables, that is, the expenditure variables denoted in Eq (4). This latent variable property is maintained after transforming the dependent variables with the inverse hyperbolic sine method, Eq (5). Zero observations reflect the decision by households to not make food and/or beverage purchases over the course of at least one calendar year in a particular store outlet.


$$\begin{array}{l} {{EX}_{ht}^{k}}^{*}={EX}_{ht}^{k}\;if\;{EX}_{ht}^{k}>0\\ {{EX}_{ht}^{k}}^{*}=0\;if\;{EX}_{ht}^{k}=0 \end{array}$$ (4)

$$\begin{array}{l} {{\bar{EX}}_{ht}^{k}}^{*}={\bar{EX}}_{ht}^{k}\;if\;{\bar{EX}}_{ht}^{k}>0\\ {{\bar{EX}}_{ht}^{k}}^{*}=0\;if\;{\bar{EX}}_{ht}^{k}=0 \end{array}$$ (5)

Our empirical model for each of the respective six store types *k* at year *t* for household *h* is described in Eqs (6) through (8). We start from the basic random effect model described in Eq (6). ${{\bar{EX}}_{ht}^{k}}^{*}$ is the annual food and beverage expenditure of household *h* at year *t* for store type *k*, censored at zero. ${{\bar{EX}}_{h,t-1}^{k}}^{*}$ is a one-year lagged dependent variable that captures the dynamic spending behavior of consumers, and $c_{h}^{k}$ is a random effect term associated with the household’s unobserved heterogeneity. $\varepsilon_{ht}^{k}$ is the idiosyncratic error term.

Producing consistent estimators with lagged dependent variables in the random effect Tobit model, controlling for the initial condition, is critical. Wooldridge [58] suggested a general and tractable approach to overcome the initial condition problem. The initial condition problem occurs when initial value of stochastic process is not observed. For example, consider following equation: $Y_{it}=\alpha +\beta Y_{it-1}+c_{i}+\varepsilon_{it}$. This equation contains a lagged dependent variable as a covariate. If we recursively rewrite this equation, then we can finally derive *Y*_*it*_ with *Y*_*i*0_ as a covariate. That said, defining *Y*_*i*0_ the initial value of the stochastic process is a difficult task. Wooldridge [58] proposed a conditional maximum likelihood estimator that approximates the unobserved heterogeneity term, *c*_*i*_, with the use of the initial observation, $y_{i,0}^{m}$, of the dataset and exogenous variables, $d_{i}^{m}$, to overcome the initial condition problem. $y_{i,0}^{m}$ is the initial observation of the value of household expenditure on food and beverages in store outlet m in 2011, the initial calendar year of the data used in this analysis.

Following Chamberlain [59], we assume that the unobserved heterogeneity term, $c_{h}^{k}$, has a distribution conditional on time-averaged continuous explanatory variables ($d_{h}^{k}$) and the value of the latent dependent variable in the initial time period (2011) of our sample (${{\bar{EX}}_{h,0}^{k}}^{*}$). In constructing the correlated random effect model, we only averaged continuous explanatory variables that are time-varying for each household. For example, for household income, we construct time-averaged variable by calculating ${\bar{in}}_{h}=\sum_{t=2011}^{2015}{in}_{ht}$, where *in*_*ht*_ corresponds to annual income for household *h* in year *t* and *t* is time period 2011 to 2015.

Then, upon substitution of the unobserved heterogeneity term, Eq (7), into the base random effect model, Eq (6), we subsequently derive Eq (8), the correlated random effect model. Because we assume, $u_{h}^{k}$, the error term in the unobserved heterogeneity function (Eq (7)), is random, and Eq (8) corresponds to a random effect model. The respective distributions of the error terms are given in Eq (9).

$z_{ht}^{k}$ is a vector of explanatory variables including the logarithm of household annual income, household size (number of household members), and number of club, convenience, drug, supercenter, and grocery stores within the household’s zip code area as well as indicator (dummy) variables related to age, education, race/ethnicity of the household head, urban/rural delineation, and region. In our correlated random effect model specification, we assume that $u_{h}^{k}$ follows a standard normal distribution with variance $\sigma_{u}^{2,k}$. In Eq (8), we can treat $u_{h}^{k}$ as a random effect. This term corresponds to household unobserved heterogeneity. To estimate this model, we employ the econometrics software package STATA version 15 using the command xttobit.


$${{\bar{EX}}_{ht}^{k}}^{*}=\alpha z_{ht}^{k}+\rho {{\bar{EX}}_{h,t-1}^{k}}^{*}+c_{h}^{k}+\varepsilon_{ht}^{k}$$ (6)

$$c_{h}^{k}=\theta {{\bar{EX}}_{h,0}^{k}}^{*}+\vartheta d_{h}^{k}+u_{h}^{k}$$ (7)

$${{\bar{EX}}_{ht}^{k}}^{*}=\alpha z_{ht}^{k}+\rho {{\bar{EX}}_{h,t-1}^{k}}^{*}+\theta {{\bar{EX}}_{h,0}^{k}}^{*}+\vartheta d_{h}^{k}+u_{h}^{k}+\varepsilon_{ht}^{k}$$ (8)

$$with\;\varepsilon_{ht}^{k}∼N(0,\;\sigma_{\varepsilon }^{2,k}),u_{h}^{k}∼N(0,\sigma_{u}^{2,k})$$ (9)

We address habitual spending behavior by adding a lagged dependent variable to the model. The coefficient of this variable, *ρ*, should be between 0 and 1. Statistical significance of this coefficient also confirms the existence of habitual spending behavior at certain store types. We jointly test significance of coefficients *θ* and *ϑ* using the Wald test to empirically check for household heterogeneity as denoted in Eq (7). Likelihood ratio tests also are performed to compare the panel data model and the pooled data model. Another likelihood ratio test is designed to compare the correlated random effect model and the simple random effect model. The pooled Tobit model is given by: ${{\bar{EX}}_{ht}^{k}}^{*}=\alpha z_{ht}^{k}+\rho {{\bar{EX}}_{h,t-1}^{k}}^{*}+\theta {{\bar{EX}}_{h,0}^{k}}^{*}+\vartheta d_{h}^{k}+\upsilon_{ht}^{k}$ which ignores the panel data structure. As discussed previously, the correlated random effect model permits the correlation among explanatory variables and unobserved heterogeneity, but the simple random effect model does not.

Eq (10) shows the conditional expectation of the original (untransformed) dependent variables. $E[{EX}_{ht}^{k}|z_{ht}^{k},{EX}_{ht}^{k}>0]$ is conditional expectation when household food expenditure is greater than zero. The function Φ is the cumulative normal distribution function with mean zero and variance $\sigma_{\varepsilon }^{2,k}+\sigma_{u}^{2,k}.\;{EX}_{ht}^{k}$ refers to the original dependent variables, untransformed annual household expenditures on food and beverages by store format, *k*. $z_{ht}^{k}$ is a vector of explanatory variables associated with continuous and binary variables as elements, and $\beta_{h}^{k}$ refers to the estimated coefficients of the structural parameters.


$$E[{EX}_{ht}^{k}|z_{ht}^{k},{EX}_{ht}^{k}>0]=\frac{1}{2}\exp\left(\frac{\sigma_{\varepsilon }^{2,k}+\sigma_{u}^{2,k}}{2}\right)*\left[\exp\left(z_{ht}^{k}\beta_{h}^{k}\right)\{\frac{\Phi (\frac{\sigma_{\varepsilon }^{2,k}+\sigma_{u}^{2,k}+z_{ht}^{k}\beta_{h}^{k}}{\sqrt{\sigma_{\varepsilon }^{2,k}+\sigma_{u}^{2,k}}})}{1-\Phi (\frac{-z_{ht}^{k}\beta_{h}^{k}}{\sqrt{\sigma_{\varepsilon }^{2,k}+\sigma_{u}^{2,k}}})}\}-\exp\left(-z_{ht}^{k}\beta_{h}^{k}\right)\{\frac{1-\Phi (\frac{\sigma_{\varepsilon }^{2,k}+\sigma_{u}^{2,k}-z_{ht}^{k}\beta_{h}^{k}}{\sqrt{\sigma_{\varepsilon }^{2,k}+\sigma_{u}^{2,k}}})}{\Phi (\frac{z_{ht}^{k}\beta_{h}^{k}}{\sqrt{\sigma_{\varepsilon }^{2,k}+\sigma_{u}^{2,k}}})}\}\right]$$ (10)

### Marginal effects

Marginal effects refer to changes in the dependent variables (expenditures) attributed to unit changes in the continuous explanatory variables. For discrete explanatory variables, marginal effects refer to changes in expenditures relative to base or reference categories. If the explanatory variables are continuous variables, we can take derivative with respect to these variables to obtain marginal effects. In Eq (11), we provide the expression of the marginal effects for continuous variables. ϕ is probability density function with mean zero and variance $\sigma_{\varepsilon }^{2,k}+\sigma_{u}^{2,k}$. The notations for Eq (11) are similar to the notations in Eq (10).


$$\begin{array}{l} \frac{\partial E[{EX}_{ht}^{k}|X_{ht}^{k},{EX}_{ht}^{k}>0]}{\partial X_{ht}^{k}}=\beta_{h}^{k}\frac{1}{2}\exp\left(\frac{\sigma_{\varepsilon }^{2,k}+\sigma_{u}^{2,k}}{2}\right)\left[\exp\left(X_{ht}^{k}\beta_{h}^{k}\right)\{\frac{\Phi (\frac{\sigma_{\varepsilon }^{2,k}+\sigma_{u}^{2,k}+X_{ht}^{k}\beta_{h}^{k}}{\sqrt{\sigma_{\varepsilon }^{2,k}+\sigma_{u}^{2,k}}})}{1-\Phi (\frac{-X_{ht}^{k}\beta_{h}^{k}}{\sqrt{\sigma_{\varepsilon }^{2,k}+\sigma_{u}^{2,k}}})}\}-\frac{1}{\sqrt{\sigma_{\varepsilon }^{2,k}+\sigma_{u}^{2,k}}}\{\frac{\phi (\frac{\sigma_{\varepsilon }^{2,k}+\sigma_{u}^{2,k}+X_{ht}^{k}\beta_{h}^{k}}{\sqrt{\sigma_{\varepsilon }^{2,k}+\sigma_{u}^{2,k}}})(1-\Phi (\frac{-X_{ht}^{k}\beta_{h}^{k}}{\sqrt{\sigma_{\varepsilon }^{2,k}+\sigma_{u}^{2,k}}}))-\Phi (\frac{\sigma_{\varepsilon }^{2,k}+\sigma_{u}^{2,k}+X_{ht}^{k}\beta_{h}^{k}}{\sqrt{\sigma_{\varepsilon }^{2,k}+\sigma_{u}^{2,k}}})\phi (\frac{-X_{ht}^{k}\beta_{h}^{k}}{\sqrt{\sigma_{\varepsilon }^{2,k}+\sigma_{u}^{2,k}}})}{{\left(1-\Phi (\frac{-X_{ht}^{k}\beta_{h}^{k}}{\sqrt{\sigma_{\varepsilon }^{2,k}+\sigma_{u}^{2,k}}})\right)}^{2}}\}\right]\\ if\;z_{ht}^{k}\;corresponds\;to\;countinuos\;variables \end{array}$$ (11)

In Eq (12), we represent the marginal effects of binary explanatory variables. We calculate these marginal effects by taking the difference between the conditional expectation when $z_{ht}^{k}=1$ and when $z_{ht}^{k}=0$.


$$\begin{array}{l} \frac{\partial E[{EX}_{ht}^{k}|z_{ht}^{k},{EX}_{ht}^{k}>0]}{\partial z_{ht}^{k}}=E[{EX}_{ht}^{k}|z_{ht}^{k}=1,E_{ht}^{k}>0]-E[{EX}_{ht}^{k}|z_{ht}^{k}=0,{EX}_{ht}^{k}>0]\\ if\;z_{ht}^{k}\;\mathrm{corresponds}\;\mathrm{to}\;\mathrm{binary}\;\mathrm{variables} \end{array}$$ (12)

We employ a lagged dependent variable and the logarithm of household income in our explanatory variable set. Marginal effects for these variables need to be treated with care. The marginal effect associated with the lagged dependent variables is given in Eq (13).


$$\frac{\partial E[{EX}_{ht}^{k}|X_{ht}^{k},{EX}_{ht}^{k}>0]}{\partial {EX}_{ht}^{k}}=\frac{\partial E[{EX}_{ht}^{k}|X_{ht}^{k},{EX}_{ht}^{k}>0]}{\partial {{\bar{EX}}_{h,t-1}^{k}}^{*}}*\frac{\partial {{\bar{EX}}_{h,t-1}^{k}}^{*}}{\partial {EX}_{ht}^{k}}$$ (13)

To derive the marginal effect for income, we simply divide Eq (11) by income.

Following McDonald and Moffitt [60], for the Tobit model, the derivative of the unconditional expectation with respect to explanatory variables can be decomposed into two parts: (1) the conditional marginal effect times the probability of non-zero household food expenditures at the various store outlets and (2) the conditional expectation times the change in probability of non-zero expenditures due to unit changes in the explanatory variables.


$$\frac{\partial E[{{EX}_{ht}^{k}}^{*}|z_{ht}^{k}]}{\partial z_{ht}^{k}}=\frac{\partial P[{{EX}_{ht}^{k}}^{*}>0|z_{ht}^{k}]}{\partial z_{ht}^{k}}*E[{{EX}_{ht}^{k}}^{*}|z_{ht}^{k},{{EX}_{ht}^{k}}^{*}>0]+P[{{EX}_{ht}^{k}}^{*}>0|z_{ht}^{k}]*\frac{\partial E[{{EX}_{ht}^{k}}^{*}|z_{ht}^{k},{{EX}_{ht}^{k}}^{*}>0]}{\partial z_{ht}^{k}}$$ (14)

We adopt this decomposition to explore the effects of explanatory variables on the probability of households spending at various store formats, $\frac{\partial P({{EX}_{ht}^{k}}^{*}>0|z_{ht}^{k})}{\partial z_{ht}^{k}}$ in Eq (14) as well as to explore the effects of explanatory variables on the magnitude of spending at particular store formats, $\frac{\partial E({{EX}_{ht}^{k}}^{*}|z_{ht}^{k},Y>0)}{\partial z_{ht}^{k}}$ in Eq (14). Note that the effects on the magnitude of spending for various store formats are the same as the conditional expectation previously described in Eq (10). With the assumption of a Tobit model, the probability is given by the linear combination of estimated coefficients associated with $z_{ht}^{k}$ for store outlet k. The change in the probability is given the probability density function times the estimated coefficients divided by the estimate of the variance of the normal distribution.

With the assumption of a Tobit model, the probability is given by the linear combination of estimated coefficients associated with z_ht_ for store outlet k. The change in the probability is given the probability density function times the estimated coefficients divided by the estimate of the variance of the normal distribution.

We calculate the marginal effects associated with Eq (10) with the use of the software package STATA15. Standard errors of the marginal effects are obtained using the delta method, as these are nonlinear combinations of coefficients and the data [49].

### Data

The source of the data for this study is the Nielsen Homescan Panel covering the period between 2011 and 2015, the most recent data available to us at the time of this analysis. Volpe, Jaenicke, and Chenarides [27] also use the Nielsen Homescan Panel for the period between 2004 and 2010. As such, we extend the time period of coverage concerning expenditures made by U.S. households at various store outlets. Hence, our analysis not only allows us to check on robustness of findings from the literature but also serves as a reference for future studies using more recent data.

Nielsen collects weekly surveys from more than 60,000 panelists every year in the entire United States. The Homescan data contain detailed information about quantities purchased and corresponding expenditures made by household by Universal Product Code (UPC) and by store type, Also, the Nielsen Homescan data incorporate a plethora of socio-demographic variables to account for household characteristics. We focus attention on *annual* expenditures made by households for all food and beverage items. Finally, we consider those households that participate in each of the five years over the period between 2011 and 2015. In our balanced panel, 28,109 households participate in the survey for the five-year period from 2011 to 2015. Hence, the total number of observations available in our study is 140,545.

In Table 1, we report the unconditional and conditional means and standard deviations of household food and beverage expenditure expressed in dollars by store type. The term conditional corresponds to only those expenditure values above zero. On the other hand, the term unconditional refers to zero values as well as non-zero expenditure values. Not unexpectedly, household spending for food and beverages is highest in grocery stores, and lowest in dollar stores.

**Table 1:** Unconditional and conditional means and standard deviations of household food and beverage expenditure by store type, Nielsen Panel data 2011 to 2015^a^.

Store Type	Unconditional	Conditional
Club	573 ^a^	1,001
	(1,018)	(1,176)
Convenience	81	267
	(345)	(588)
Dollar	74	114
	(189)	(226)
Grocery	2,177	2,197
	(1,750)	(1,745)
Drug	259	332
	(557)	(575)
Discount	1,004	1,072
	(1,302)	(1,318)
Across All Store Outlets	4,162	4,982

^a^ All values are expressed in terms of dollars, and standard deviations are in parentheses.

Not all households purchase food and beverages at all store outlets even over a calendar year. Therefore, zero values are evident in household expenditures for food and beverages across the respective store types. The number of zero observations and the degree of censoring of household expenditures on food and beverages is exhibited in Table 2. The degree of censoring is defined as the number of zero observations times 100 divided by the number of observations.

**Table 2:** Number of zero values and degree of censoring for household food and beverage expenditures by store type, Nielsen Panel data 2011 to 2015.

Store Type	Number of Zero Values/Degree of Censoring
Club	60,148^a^ (42.8%)^b^
Convenience	98,239 (70.0%)
Dollar	49,552 (35.3%)
Grocery	1,286 (0.9%)
Drug	28,067 (20.0%)
Discount	8,802 (6.3%)
Total number of observations	140,545

^a^ Number of zero observations associated with household food and beverage expenditures.

^b^ Degree of censoring expressed as a percentage.

(Degree of censoring = number of zero observations*100/total number of observations)

The degree of censoring is greatest for convenience stores at roughly 70 percent, and the degree of censoring is lowest in grocery stores at approximately 1 percent. In discount stores, the degree of censoring is on the order of 6 percent; in drug stores, the degree of censoring is on the order of 20 percent. In dollar stores, the magnitude of censoring is slightly more than 35 percent. Finally, for club stores, the degree of censoring is nearly 43 percent.

Similar to Kyureghian and Nayga [15], we use store density data to account for the retail environment. However, we examine store density by zip code, which represents smaller residential areas rather than by county level as was done by Kyureghian and Nayga [15]. These variables were obtained from Business Pattern Data (BPD hereafter) produced by the U.S. Census Bureau. The BPD contains data representing the number of stores categorized by the North American Industry Classification System (NAICS). From these data, we obtained counts concerning four types of store outlets (grocery stores and supercenters, NAICS code 445110; warehouse club stores, NACIS code 452910; convenience stores, NAICS code 445120; and drug stores, NAICS code 446110). The data from the Nielsen Homescan Panel are available by zip code; consequently, we are able to augment the Nielsen Homescan Panel with the respective counts of store outlets from BPD. A shortcoming in this augmentation process is that the classifications of store formats from the Nielsen Homescan Panel and Business Pattern Data are different. Nevertheless, we provide a viable proxy for the retail environment based on counts of store outlets from BPD.

In order to identify differences in food and beverage expenditures by store outlets between households who live in urban and rural areas, we form urban and rural indicator (dummy) variables. Our dummy variables correspond to the six-category urban and rural classification scheme developed by the National Center for Health Statistics (NCHS). In our study, we form three dummy variables that represent category 1 through category 6 of the NCHS classification scheme. The dummy variable URBAN corresponds to NCHS urban and rural classification categories 1 and 2, the most densely populated areas, typically metropolitan areas. The dummy variable RURAL corresponds to NCHS urban and rural classification categories 5 and 6, rural areas with the least dense population. The dummy variable not classified as urban or rural corresponds to NCHS urban and rural classification categories 3 and 4. Then, we aligned these indicator variables with our Nielsen Homescan data based on zip code. The use of the NCHS classification scheme affords a richer consideration of the role of urban and rural areas in influencing household food and beverage expenditures by store outlet.

As exhibited in Table 3, the means and standard deviations of explanatory variables in the model are presented. The average number of grocery stores by zip code is slightly more than five. The average number of drug stores by zip code in this analysis is nearly four across the board. Similarly, on average the number of convenience stores by zip code is between two and three for each of the respective samples. Finally, the average number of club stores by zip code is between zero and one, again across the board. In sum, with respect to the number of store outlets by zip code, the average number of club stores, convenience stores, supercenters and grocery stores, and drug stores does not vary much among the entire sample. More than half of the sample households live in urban areas, and 15 percent live in rural areas. The base category or reference category with respect to degree of urbanization is the ‘not urban or not rural’ category.

**Table 3:** Means and standard deviations of explanatory variables in the Tobit random effect model.

Variable Name	Variable Description	Mean/Standard Deviation
**Continuous Variables**		
Household income	Household income	$53,589 ($26,601)
Household size	Number of household members	2.17 (1.15)
Cu	NAICS code 452910, # of warehouse club stores by zip code	0.54 (0.80)
Cv	NAICS code 445120, # of convenience stores by zip code	2.36 (2.98)
Sg	NAICS code 445110, # of supercenters and grocery stores by zip code	5.42 (7.19)
Dr	NAICS code 446110, # of drug stores by zip code	3.82 (3.59)
**Degree of Urbanization**		
Urban	NCHS urban and rural classification categories 1 and 2	0.54 (0.50)
Not urban* or rural	NCHS urban and rural classification categories 3 and 4)	0.30 (0.46)
Rural	NCHS urban and rural classification categories 5 and 6	0.15 (0.36)
**Age**		
Age<40	Age of household head below 40	0.01 (0.12)
40<Age<60*	Age of household head above 40 and below 60	0.18 (0.38)
Age>60	Age of household head over 60	0.82 (0.39)
**Education**		
Under high school	Household head education less than high school	0.01 (0.10)
Graduate high school	Household head is a high school graduate	0.18 (0.39)
College experience	Household head had some college education but is not a college graduate	0.28 (0.45)
Graduate* college	Household head is a college graduate	0.53 (0.50)
Variable Name	Variable Description	Mean/Standard Deviation
**Race and ethnicity**		
Nonhisp-* White	Household head is non-Hispanic white	0.82 (0.39)
Nonhisp-Black	Household head is non-Hispanic black	0.09 (0.29)
Nonhisp-Asian	Household head is non-Hispanic Asia.	0.03 (0.16)
Nonhisp-Other	Household head is non-Hispanic other	0.02 (0.14)
Hisp	Household head is Hispanic	0.04 (0.21)
**Region**		
Ne	Household located in the New England region (Connecticut, Maine, Massachusetts, New Hampshire, Rhode Island, and Vermont)	0.05 (0.21)
Ma	Household located in the Middle Atlantic region, (New Jersey, New York, and Pennsylvania)	0.13 (0.34)
Enc	Household located in East North Central region (Illinois, Indiana, Michigan, Ohio, and Wisconsin)	0.18 (0.39)
Wnc	Household located in the West North Central region (Iowa, Kansas, Minnesota, Missouri, Nebraska, North Dakota, and South Dakota)	0.09 (0.29)
Sa	Household located in the South Atlantic region (Delaware, Florida, Georgia, Maryland, North Carolina, South Carolina, Virginia, District of Columbia, and West Virginia)	0.20 (0.40)
Esc	Household located in East South Central region (Alabama, Kentucky, Mississippi, and Tennessee)	0.06 (0.23)
Wsc	Household located in the West South Central region (Arkansas, Louisiana, Oklahoma, and Texas)	0.10 (0.30)
Mt	Household located in the Mountain region (Arizona, Colorado, Idaho, Montana, Nevada, New Mexico, Utah, and Wyoming)	0.07 (0.26)
Pac*	Household located in the Pacific region (Alaska, California, Hawaii, Oregon, and Washington)	0.12 (0.33)
Number of observations		140,545

Standard deviations are in parentheses.

Superscript

* associated with the variable name indicates the base category or reference category.

Additionally, in Table 3 we present descriptive statistics concerning socio-demographic variables, namely household income, household size, age, education level, race/ethnicity of the household head, and region in which the household is located. Household income is reported by ranges in the Nielsen Homescan Panel. Similar to previous studies, we take the midpoint of each household income range as the income level of the household [15, 61, 62]. The mean value of household income for the entire sample is $53,589. The average household size for the entire sample is 2.17 members.

We employ three classifications of the age of the household head, less than 40, between 40 and 60, and over 60. The proportion of households whose heads are less than 40 is roughly 1 percent; the proportion of households whose heads are between 40 and 60 is 18 percent; and the proportion of households whose heads are over 60 is 82 percent. Across the respective data samples, the number of households whose heads are less than 40 is around 1 percent. The data concerning age of the household head unequivocally are skewed toward older household heads. The base category is age of the household head between 40 and 60.

We consider four categories concerning the level of education of the household head—less than high school, high school graduate, some college experience, and college graduate. To illustrate, the proportion of households whose heads have a college degree is 53 percent. Further, the proportion of households wherein the highest level of education is a high school degree is 18 percent. Very few household heads in the respective data samples have less than a high-school education. The vast majority of household heads in the respective data samples have at least some college-level educations. The base category of education level corresponds to household heads with a college degree.

We employ five joint classifications of the race and ethnicity of the household head—non-Hispanic white, non-Hispanic black, non-Hispanic Asian, non-Hispanic other, and Hispanic. The proportion of households whose heads are non-Hispanic white is 82 percent; the proportion of households whose heads are non-Hispanic black is 9 percent; the proportion of households whose heads are non-Hispanic Asian is 3 percent; the proportion of households whose heads are non-Hispanic other races is 2 percent; and the proportion of Hispanic household heads is 4 percent. The base category of race/ethnicity is non-Hispanic white households.

We rely on nine categories concerning the region in which the household is located (1) New England; (2) Middle Atlantic; (3) East North Central; (4) West North Central; (5) South Atlantic; (6) East South Central; (7) West South Central; (8) Mountain; and (9) Pacific. Across the board, roughly 20 percent of the households reside in the South Atlantic region, 18 percent reside in the East North Central region, 13 percent in the Middle Atlantic region, 12 percent in the Pacific region, 10 percent in the West South-Central region, 9 percent in the West North Central region, 7 percent in the Mountain region, 6 percent in the East South-Central region, and 5 percent in the New England region. The reference category is the Pacific region.

### Potential endogeneity of the retail environment variables

There is a debate in the literature as to whether the retail environment variables are endogenous. This issue is important due to the fact that the endogeneity of explanatory variables leads to inconsistent parameter estimates. The endogeneity issue was addressed in several studies [15, 63–66]. On the other hand, Currie et al. [67] and Taylor and Villas-Boas [24] argued that the endogeneity of retail environment variables does not lead to bias or inconsistency of parameter estimates.

Although previous works recognized potential endogeneity issues regarding retail environment variables, those studies just assumed the presence of endogeneity and estimated models with instrument variables. In this study, we formally test whether the retail environment variables suffer from the endogeneity problem. To account for the retail environment, we use variables that represent the number of stores, a metric of store density, by store type in the zip code area in which the household is located. As mentioned previously, we only incorporate four categories of store types, namely supercenters and grocery stores, club stores, drug stores, and convenience stores from the BPD data. Then, we test exogeneity of those four variables in each of the equations pertaining to the six store types. To carry out the Hausman test of endogeneity, we initially estimate each retail environment variable as a function of the remaining explanatory variables. Consistent with Hausman [68] we incorporate the residuals from the first-stage estimation results in the full model. Subsequently, we test the null hypothesis that the coefficients associated with these residuals in the respective equations are all equal to zero; this null hypothesis is tantamount the exogeneity of the respective retail environment variables. A rejection of this null hypothesis then is statistical evidence that the set of retail environment variables are endogenous.

Results based on the Hausman test presented in Table 4 indicate the lack of evidence of endogeneity of the retail environment variables. These results are consistent with the assumption of the lack of endogeneity of retail environment variables made by Currie et al. [67] and Taylor and Villas-Boas [24]. However, unlike previous studies, we provide statistical evidence to support the claim that the set of retail environment variables indeed are exogenous.

**Table 4:** Results of the Hausman endogeneity Chi-Squared tests associated with the retail environment variable^sa^.

Model	Club	Convenience	Dollar	Discount	Grocery	Drug
Chi-squared statistic	1.60	8.12	0.50	0.34	3.45	4.41
p-value	0.80	0.09	0.97	0.99	0.49	0.35

^a^ Chi-squared tests each with four degrees-of-freedom.

## Results

Maximum likelihood estimates of the respective parameters and standard errors in the various models are obtained with the use of the software package STATA Version 15. As shown in Table 5, we provide the parameter estimates, associated p-values, likelihood ratio and Wald tests, and goodness-of-fit metrics for the dynamic correlated random effect Tobit model. Additionally, in Table 6, we provide the marginal effects. In this study, we adopt a level of significance of 0.01 because of the sizable number of observations.

**Table 5:** Maximum likelihood parameter estimates and the associated p-values for the explanatory variables.

Explanatory Variable	Grocery	Discount	Club	Dollar	Convenience	Drug
Lagged dependent variable	0.669*	0.435*	0.509*	0.381*	0.549*	0.402*
	(0.006)	(0.005)	(0.006)	(0.005)	(0.009)	(0.005)
Income	0.000	0.023	0.059	-0.042	0.057	0.003
	(0.007)	(0.013)	(0.029)	(0.019)	(0.048)	(0.020)
Household size	0.003	0.027*	0.060*	0.032*	-0.019	-0.019*
	(0.002)	(0.004)	(0.010)	(0.007)	(0.016)	(0.007)
Age <40	0.008	-0.007	-0.116	-0.058	0.300	0.055
	(0.016)	(0.035)	(0.087)	(0.059)	(0.132)	(0.056)
Age >60	0.017*	-0.059*	0.074	0.080*	-0.018	0.095*
	(0.005)	(0.012)	(0.030)	(0.020)	(0.047)	(0.019)
Urban	0.012	-0.097*	-0.012	-0.103*	-0.397*	0.061*
	(0.005)	(0.011)	(0.030)	(0.020)	(0.045)	(0.018)
Rural	-0.030*	0.083*	-0.459*	0.105*	-0.052	-0.123*
	(0.006)	(0.015)	(0.042)	(0.027)	(0.060)	(0.025)
Under high school	-0.047	0.119*	0.001	0.173	-0.196	-0.192*
	(0.019)	(0.042)	(0.111)	(0.068)	(0.168)	(0.067)
Graduate high school	-0.031*	0.085*	-0.010	0.191*	-0.036	-0.062*
	(0.006)	(0.013)	(0.034)	(0.022)	(0.051)	(0.021)
College experienced	-0.009	0.047*	0.005	0.131*	0.098	0.003
	(0.005)	(0.010)	(0.026)	(0.017)	(0.040)	(0.016)
Non-Hispanic black	-0.034*	0.107*	0.322*	0.332*	0.326*	0.146*
	(0.007)	(0.018)	(0.046)	(0.031)	(0.069)	(0.028)
Non-Hispanic Asian	-0.041*	-0.018	0.210*	-0.054	-0.264	-0.097
	(0.013)	(0.030)	(0.075)	(0.054)	(0.128)	(0.048)
Non-Hispanic other	-0.034	0.070	0.080	0.151*	0.483*	0.034
	(0.014)	(0.031)	(0.075)	(0.051)	(0.117)	(0.048)
Hispanic	-0.013	0.083*	0.131	0.210*	0.247*	0.099*
	(0.010)	(0.023)	(0.058)	(0.040)	(0.092)	(0.037)
Number of Club stores	-0.028*	0.012	0.136*	-0.005	-0.060	-0.107*
	(0.007)	(0.014)	(0.029)	(0.021)	(0.052)	(0.021)
Number of Convenience stores	0.001	-0.003	-0.002	0.002	0.010	0.003
	(0.002)	(0.003)	(0.007)	(0.005)	(0.012)	(0.005)
Explanatory Variable	Grocery	Discount	Club	Dollar	Convenience	Drug
Number of Grocery stores and Supercenters	0.003	-0.008*	0.006	-0.003	-0.009	0.001
	(0.001)	(0.002)	(0.006)	(0.004)	(0.010)	(0.004)
Number of Drug stores	0.002	0.002	-0.009	-0.008	0.027	0.006
	(0.002)	(0.004)	(0.008)	(0.006)	(0.015)	(0.006)
New England	0.032*	-0.062	-0.363*	-0.094	0.406*	0.176*
	(0.011)	(0.027)	(0.071)	(0.049)	(0.111)	(0.043)
Middle Atlantic	0.027*	-0.032	-0.433*	0.125*	1.183*	0.058
	(0.008)	(0.020)	(0.052)	(0.035)	(0.081)	(0.032)
East North Central	0.029*	-0.042	-0.196*	0.034	0.593*	0.037
	(0.008)	(0.019)	(0.049)	(0.033)	(0.077)	(0.030)
West North Central	-0.019	0.118*	-0.235*	-0.068	1.387*	-0.064
	(0.009)	(0.022)	(0.059)	(0.039)	(0.089)	(0.036)
South Atlantic	0.006	0.086*	-0.251*	0.125*	0.773*	0.074
	(0.008)	(0.018)	(0.047)	(0.033)	(0.076)	(0.029)
East South Central	0.011	0.091*	-0.303*	0.210*	0.234	0.022
	(0.011)	(0.026)	(0.068)	(0.045)	(0.106)	(0.041)
West South Central	0.015	0.095*	-0.211*	0.065	0.386*	-0.028
	(0.009)	(0.021)	(0.056)	(0.038)	(0.089)	(0.034)
Mountain	0.015	0.060	-0.070	-0.048	0.733*	-0.111*
	(0.010)	(0.023)	(0.059)	(0.041)	(0.095)	(0.037)
Constant	0.491*	0.675*	-6.118*	1.070*	-4.208*	-0.934*
	(0.041)	(0.095)	(0.260)	(0.170)	(0.377)	(0.152)
Initial value	0.224*	0.460*	0.686*	0.657*	0.849*	0.526*
	(0.005)	(0.005)	(0.007)	(0.006)	(0.011)	(0.005)
*σ*_*u*_	0.217*	0.649*	1.802*	1.220*	2.448*	1.062*
	(0.005)	(0.007)	(0.017)	(0.011)	(0.028)	(0.011)
*σ*_*e*_	0.586*	1.114*	2.107*	1.532*	3.035*	1.668*
	(0.002)	(0.003)	(0.007)	(0.004)	(0.013)	(0.004)
LR *χ*^2^ test of the Correlated Random Effect Tobit Model vs the Pooled Tobit Model	378.9*	2,675.5*	5,227.7*	6,472.5*	4,288.8*	3,540.3*
LR *χ*^2^ test of the Correlated Random Effect Tobit Model vs Random Effect Tobit Model	2,748.9*	9,085.7*	9,062.9*	12,033.9*	6,808.6*	9,791.9*
Wald *χ*^2^ test	1,965.2*	9,730.3*	10,690.9*	12,334.3*	5,760.3*	10,201.6*
Pseudo *R*^2^	0.323	0.233	0.233	0.221	0.212	0.215
V-Z *R*^2^	0.778	0.702	0.749	0.678	0.542	0.603
Number of Observations	140,545	140,545	140,545	140,545	140,545	140,545
Number of Households	28,109	28,109	28,109	28,109	28,109	28,109

*p<0.01Numbers in parentheses correspond to standard errors.

**Table 6:** Conditional marginal effects of household food and beverage expenditures and marginal effects associated with the probability of purchasing by store type.

	Grocery		Discount		Club		Dollar		Convenience		Drug	
	Probability to purchase	Expenditure	Probability to purchase	Expenditure	Probability to purchase	Expenditure	Probability to purchase	Expenditure	Probability to purchase	Expenditure	Probability to purchase	Expenditure
Household income	0.000	0.000	0.0003	0.016	0.004	0.035	-0.005	-0.017	0.004	0.225	0.000	0.002
	(0.000)	(0.005)	(0.0002)	(0.009)	(0.002)	(0.017)	(0.002)	(0.008)	(0.003)	(0.190)	(0.001)	(0.002)
Household size	0.000	5.802	0.0003*	19.397*	0.004*	21.054*	0.004*	0.991*	-0.001	-6.401	-0.001*	-4.196*
	(0.000)	(3.312)	(0.0001)	(3.042)	(0.001)	(3.678)	(0.001)	(0.214)	(0.001)	(5.371)	(0.000)	(1.498)
Age<40	0.000	14.836	-0.0001	-4.791	-0.0075	-40.738	-0.0063	-1.781	0.0203	98.527	0.0028	12.384
	(0.000)	(28.944)	(0.0004)	(25.412)	(0.0056)	(30.632)	(0.0064)	(1.809)	(0.0089)	(43.760)	(0.0029)	(12.660)
Age>60	0.000*	29.658*	-0.0007*	-42.166*	0.0048	25.921	0.0087*	2.437*	-0.0012	-6.039	0.0049*	21.417*
	(0.000)	(9.634)	(0.0001)	(8.724)	(0.0019)	(10.562)	(0.0022)	(0.624)	(0.0032)	(15.466)	(0.001)	(4.366)
Urban	0.000	21.073	-0.0012*	-69.750*	-0.0008	-4.251	-0.0111*	-3.133*	-0.0268*	-130.382*	0.0032*	13.872*
	(0.000)	(8.518)	(0.0001)	(8.258)	(0.0019)	(10.480)	(0.0022)	(0.616)	(0.0031)	(17.315)	(0.0009)	(4.122)
Rural	-0.000*	-54.280*	0.0010*	59.642*	-0.0297*	-161.137*	0.0113*	3.193*	-0.0035	-17.216	-0.0063*	-27.790*
	(0.0000)	(11.595)	(0.0002)	(11.141)	(0.0027)	(15.793)	(0.0029)	(0.825)	(0.0041)	(19.859)	(0.0013)	(5.667)
Less than high school	-0.000	-83.340	0.0014*	85.207*	0.0000	0.223	0.0188	5.297	-0.0133	-64.520	-0.0099*	-43.417*
	(0.000)	(33.734)	(0.0005)	(30.384)	(0.0072)	(39.050)	(0.0074)	(2.081)	(0.0114)	(55.478)	(0.0034)	(15.167)
High school graduate	-0.000*	-55.831*	0.0010*	60.830*	-0.0007	-3.650	0.0207*	5.834*	-0.0025	-11.922	-0.0032*	-13.976*
	(0.000)	(10.130)	(0.0002)	(9.411)	(0.0022)	(11.786)	(0.0024)	(0.678)	(0.0035)	(16.863)	(0.0011)	(4.698)
College experienced	0.000	-16.898	0.0006*	33.611*	0.0003	1.858	0.0143*	4.013*	0.0066	32.306	0.0001	0.587
	(0.000)	(8.271)	(0.0001)	(7.486)	(0.0017)	(9.034)	(0.0019)	(0.529)	(0.0027)	(13.408)	(0.0008)	(3.714)
Non-Hispanic black	-0.000*	-60.939*	0.0013*	76.712*	0.0208*	113.054*	0.0361*	10.151*	0.0220*	107.134*	0.0075*	32.938*
	(0.000)	(13.153)	(0.0002)	(12.626)	(0.003)	(16.484)	(0.0033)	(0.952)	(0.0047)	(23.956)	(0.0014)	(6.327)
Non-Hispanic Asian	-0.000*	-73.118*	-0.0002	-12.856	0.0136*	73.620*	-0.0058	-1.644	-0.0178	-86.734	-0.005	-21.991
	(0.000)	(22.648)	(0.0004)	(21.588)	(0.0049)	(26.471)	(0.0058)	(1.645)	(0.0087)	(42.508)	(0.0025)	(10.826)
Non-Hispanic other	0.000	-60.696	0.0009	50.558	0.0052	28.280	0.0164*	4.625*	0.0326*	158.542*	0.0018	7.759
	(0.000)	(24.240)	(0.0004)	(21.974)	(0.0048)	(26.294)	(0.0055)	(1.553)	(0.0079)	(39.953)	(0.0025)	(10.911)
Hispanic	0.000	-22.642	0.0010*	59.347*	0.0085	46.169	0.0228*	6.431*	0.0167*	81.235*	0.0051*	22.261*
	(0.000)	(17.703)	(0.0003)	(16.627)	(0.0038)	(20.450)	(0.0043)	(1.217)	(0.0062)	(30.862)	(0.0019)	(8.309)
	Grocery		Discount		Club		Dollar		Convenience		Drug	
	Probability to purchase	Expenditure	Probability to purchase	Expenditure	Probability to purchase	Expenditure	Probability to purchase	Expenditure	Probability to purchase	Expenditure	Probability to purchase	Expenditure
Club stores	-0.000*	-49.715*	0.0002	8.843	0.0088*	47.614*	-0.0006	-0.165	-0.004	-19.603	-0.0055*	-24.109*
	(0.0000)	(12.877)	(0.0002)	(9.903)	(0.0019)	(10.435)	(0.0023)	(0.640)	(0.0035)	(16.998)	(0.0011)	(4.819)
Convenience stores	0.000	1.583	0.0000	-2.364	-0.0001	-0.655	0.0003	0.071	0.0007	3.264	0.0001	0.574
	(0.000)	(2.921)	(0.0000)	(2.256)	(0.0004)	(2.409)	(0.0005)	(0.144)	(0.0008)	(3.914)	(0.0002)	(1.081)
Grocery stores and Supercenters	0.000	5.425	-0.0001*	-5.988*	0.0004	1.993	-0.0003	-0.093	-0.0006	-3.002	0.0001	0.288
	(0.000)	(2.310)	(0.0000)	(1.791)	(0.0004)	(1.961)	(0.0004)	(0.117)	(0.0006)	(3.161)	(0.0002)	(0.850)
Drug stores	0.000	3.289	0.0000	1.436	-0.0006	-3.044	-0.0009	-0.247	0.0018	8.979	0.0003	1.335
	(0.000)	(3.549)	(0.0000)	(2.747)	(0.0005)	(2.934)	(0.0006)	(0.179)	(0.001)	(4.857)	(0.0003)	(1.315)
Ne	0.000*	57.923*	-0.0008	-44.697	-0.0235*	-127.559*	-0.0102	-2.872	0.0274*	133.498*	0.0090*	39.749*
	(0.000)	(20.147)	(0.0003)	(19.500)	(0.0046)	(25.435)	(0.0053)	(1.493)	(0.0075)	(37.670)	(0.0022)	(9.780)
Ma	0.000*	48.799*	-0.0004	-23.331	-0.0280*	-152.223*	0.0136*	3.823*	0.0799*	388.642*	0.003	13.148
	(0.000)	(14.770)	(0.0002)	(14.299)	(0.0034)	(19.031)	(0.0038)	(1.083)	(0.0055)	(37.455)	(0.0016)	(7.168)
Enc	0.000*	51.261*	-0.0005	-30.494	-0.0127*	-68.744*	0.0037	1.053	0.0400*	194.826*	0.0019	8.275
	(0.000)	(13.762)	(0.0002)	(13.311)	(0.0032)	(17.288)	(0.0036)	(1.009)	(0.0052)	(28.526)	(0.0015)	(6.681)
Wnc	0.000	-34.301	0.0014*	84.810*	-0.0152*	-82.561*	-0.0074	-2.070	0.0936*	455.453*	-0.0033	-14.457
	(0.000)	(16.422)	(0.0003)	(15.954)	(0.0038)	(20.807)	(0.0043)	(1.205)	(0.006)	(42.433)	(0.0018)	(8.020)
Sa	0.000	11.413	0.0010*	61.508*	-0.0163*	-88.361*	0.0135*	3.805*	0.0522*	253.879*	0.0038	16.650
	(0.000)	(13.610)	(0.0002)	(13.229)	(0.0031)	(16.908)	(0.0035)	(0.999)	(0.0051)	(30.219)	(0.0015)	(6.606)
Esc	0.000	19.602	0.0011*	65.531*	-0.0196*	-106.356*	0.0228*	6.421*	0.0158	76.943	0.0011	4.997
	(0.000)	(19.085)	(0.0003)	(18.453)	(0.0044)	(24.306)	(0.0049)	(1.379)	(0.0072)	(35.345)	(0.0021)	(9.276)
Wsc	0.000	27.010	0.0012*	68.543*	-0.0137*	-74.157*	0.0071	1.998	0.0261*	126.887*	-0.0015	-6.395
	(0.000)	(15.852)	(0.0003)	(15.368)	(0.0036)	(19.686)	(0.0041)	(1.154)	(0.006)	(30.309)	(0.0017)	(7.689)
Mt	0.000	27.014	0.0007	42.932	-0.0045	-24.561	-0.0052	-1.473	0.0495*	240.857*	-0.0057*	-25.025*
	(0.000)	(17.299)	(0.0003)	(16.691)	(0.0038)	(20.910)	(0.0045)	(1.263)	(0.0064)	(35.094)	(0.0019)	(8.429)

Superscript

* indicates p-value <0.01.

Numbers in parentheses correspond to standard errors.

The marginal effect of the probability to purchase at any store outlet is given by $\frac{\partial P({{EX}_{ht}^{k}}^{*}>0|z_{ht}^{k})}{\partial z_{ht}^{k}}$, and the marginal effect of the conditional expectation of household food and beverage expenditures is expressed mathematically as $\frac{\partial E[{EX}_{ht}^{k}|z_{ht}^{k},{EX}_{ht}^{k}>0]}{\partial z_{ht}^{k}}$. All calculations pertaining to marginal effects are made at the sample means of the data.

The parameter estimates associated with the standard deviation of the random effect term $\sigma_{u}^{k}$, are statistically significant for all store types. As such, household unobserved heterogeneity plays a decisive role in food purchasing behavior. Based on likelihood ratio tests, the correlated random effect Tobit model is superior to the pooled Tobit model as well as the random effect Tobit model. The Wald test is the analogue of the conventional F-statistic in regression analysis. For each of the respective six models, the Wald tests support the contention that at least one estimated coefficient is statistically different from zero. Alternatively, the Wald tests support the hypothesis that each model explains a significant amount of variation in household food and beverage expenditures across all store outlets.

We report two different goodness-of-fit metrics to determine the degree of explanatory power associated with each of the respective correlated random effect Tobit models. The first measure, labeled as pseudo R^2^, is the square of the correlation of the ***unconditional*** expected value and the actual value of household food and beverage expenditures. As exhibited in Table 5, the pseudo R^2^ ranges from 0.212 to 0.323. Alternatively, we use the computation method to calculate the goodness-of-fit metric proposed by Veall and Zimmermann [69] (V-Z hereafter). The V-Z pseudo R^2^ statistic is the square of the correlation of the ***conditional*** expected value and the actual value of household food and beverage expenditures. As shown in Table 5, the V-Z pseudo R^2^ranges from 0.542 to 0.778. Based on these goodness-of-fit measures, the correlated random effect Tobit models explain a notable amount of the variability in household food and beverage expenditures for each store type.

### Estimation results

As exhibited in Table 5, expenditures made in the previous year are positively related to current expenditures across all store outlets. The coefficients of the lagged dependent variable are statistically significant, ranging from 0.381 (dollar stores) to 0.669 (grocery stores). As such, these results support our hypothesis of habit persistence associated with food and beverage expenditures made by households. We confirm the supposition of habitual spending across all store outlets. These results suggest that, within our data period 2011 to 2015, habitual spending behavior is a key factor in affecting nominal food and beverage expenditures across all store outlets.

Household income is not a statistically significant factor affecting household food and beverage expenditures in any of the respective store outlets. Household size is positively related to household expenditures made at discount stores, club stores, and dollar stores, but household size is negatively related to household expenditures made at drug stores. Relative to households in the 40-year-old to 60-year-old category, household expenditures made at grocery stores, dollar stores, and drug stores are higher for households 60 years of age and older. But household expenditures made at discount stores are lower for households 60 years of age and older. No statistically significant differences are evident for households less than 40 years of age and for households in the 40-year-old to 60-year-old category.

For households located in urban areas, household food and beverage expenditures are higher in drug stores, but lower in discount stores, dollar stores, and convenience stores relative to households located outside of urban and rural areas. For households located in rural areas, household food and beverage expenditures are higher in discount stores and dollar stores but lower in grocery stores, club stores, and drug stores relative to households located outside of urban and rural areas.

Relative to households who have graduated from college, households with less than a high school education spend more at discount stores but less at drug stores. Households with a high school education spend more on food and beverages at discount stores and dollar stores but less at grocery stores and drug stores. Households with some level of college experience spend more at discount stores and dollar stores.

The reference category of race indicator variables is non-Hispanic white households. Non-Hispanic black households spend more on food and beverages at discount stores, club stores, dollar stores, convenience stores, and drug stores but less at grocery stores. Non-Hispanic Asian households spend more on food and beverages at club stores but less at grocery stores. Non-Hispanic non-black and non-Asian households spend more on food and beverages at dollar stores and convenience stores. Finally, Hispanic households spend more on food and beverages at discount stores, dollar stores, convenience stores, and drug stores.

The number of club stores within the residence of households negatively impacts food and beverage expenditures made at grocery stores and drug stores but positively affects food and beverage expenditures made at club stores. The number of grocery stores and supercenters within the residence of households negatively impacts expenditures made at discount stores. On the other hand, the number of convenience stores and the number of drug stores within the residence of households are not statistically significant factors affecting expenditures made at any of the six store outlets.

Relative to households located in the Pacific region, food and beverage expenditures made by households located in the New England, Middle England and East North Central regions are higher at grocery stores. Households located in the West North Central, South Atlantic, East South Central, and West South Central regions spend greater amounts at discount stores. Except for the Mountain region, household expenditure at club stores is lower in all regions than in the Pacific region. Household expenditure at dollar stores is greater in the Middle Atlantic, South Atlantic, and East South Central regions than in the Pacific region. In all regions except the Mountain region, households spend more amount at convenience stores than do households located in the Pacific region. Households located in New England spend more for food and beverages at drug stores than do households located in the Pacific region. Without question, region is a key determinant of household food and beverage expenditures across the six store outlets.

### Conditional marginal effects

As presented in Table 6, changes in household income do not significantly affect the level of household expenditure for food and beverages. Unit increases in household size result in a $19.40 increase in household expenditures at discount stores, a $21.05 rise in household expenditures at club stores, and a $0.99 increase in household expenditures at dollar stores annually. But unit increases in household size result in a decline of $4.20 at drug stores annually, holding all other factors invariant.

Relative to households in the 40-year-old to 60-year-old category, household expenditures made at grocery stores, club stores, dollar stores, and drug stores are higher by $29.66, $25.92, $2.43, and $21.42 annually for households 60 years of age and older. Household expenditures made at discount stores are lower by $42.17 annually for households 60 years of age and older.

For households located in urban areas, household food and beverage expenditures are higher in grocery stores and in drug stores by $21.07 and $13.87 annually, but lower in discount stores, dollar stores, and convenience stores by $69.75, $3.13, and $130.38 respectively on an annual basis relative to households located outside of urban and rural areas (base category). For households located in rural areas, household food and beverage expenditures are higher in discount stores and dollar stores by $59.42 and $3.19 annually but lower in grocery stores, club stores, and drug stores by $54.28, $161.14, and $27.79 annually.

Relative to households who have graduated from college (base category), households with less than a high school education expend $85.21 more at discount stores but $43.41 less at drug stores annually. Households with a high school education spend $60.83 more and $5.83 more on food and beverages at discount stores and dollar stores but $55.83 less at grocery stores and $13.98 less at drug stores. Households with some level of college experience expend $33.61 more at discount stores and $4.01 more at dollar stores annually.

Relative to non-Hispanic white households (base category), non-Hispanic black households spend $76.71, $113.05, $10.51, $107.13, and $32.94 more on food and beverages at discount stores, club stores, dollar stores, convenience stores, and drug stores respectively, but $60.94 less at grocery stores. Asian households spend $73.62 more on food and beverages at club stores but $73.12 less at grocery stores annually. Non-Hispanic non-black and non-Asian households spend $4.63 and $158.54 more on food and beverages at dollar stores and convenience stores annually. Finally, Hispanic households spend $59.35, $6.43, $81.24, and $22.26 more on food and beverages at discount stores, dollar stores, convenience stores, and drug stores, respectively on an annual basis.

For each unit increase in the number of club stores, household expenditures made at grocery stores decline by $49.72 and expenditures made at drug stores decline by $24.11. On the other hand, for each unit increase in club stores, expenditures made at club stores increases by $47.61. For each unit increase in the number of grocery stores and supercenters, household expenditures made at discount stores decline by $5.99.

Relative to households located in the Pacific region (base category), food and beverage expenditures made by households located in the New England region are higher $57.92, $133.50, and $39.75 annually at grocery stores, convenience stores, and drug stores respectively but are lower by $127.56 at club stores; in the Middle Atlantic region, higher by $48.80, $3.82, $388.64 at grocery stores, dollar stores, and convenience stores respectively but lower by $152.22 at club stores; in the East North Central region, higher by $51.26 at grocery stores and by $194.83 at convenience stores but lower by $68.74 at club stores; in the West North Central region, higher by $84.81 at discount stores and by $455.45 at convenience stores but lower by $82.56 at club stores; in the South Atlantic region, higher by $61.51, $3.81, and $253.88 at discount stores, dollar stores, and convenience stores but lower by $88.36 at club stores; in the East South Central region, higher by $65.53 at discount stores and by $6.42 at dollar stores but lower by $106.36 at club stores; in the West South Central region, higher by $68.54 at discount stores and by $126.89 at convenience stores but lower by $74.16 at club stores; and, in the Mountain region, higher by $240.86 at convenience stores but lower by $25.03 at drug stores.

### Marginal effects associated with the probability of purchasing

As exhibited in Table 6, the probability of purchasing food and beverages at grocery stores are significantly higher for households who are greater than 60 years of age as well as households located in New England, the Mid-Atlantic, and the East North Central regions of the United States. But this probability is significantly lower for household located in rural areas, for households with at most a high school education, for non-Hispanic black households and for non-Hispanic Asian households. That said, the magnitude of the marginal effects associated with purchasing food and beverages at grocery stores albeit statically significant is negligible from a practical standpoint.

As presented in Table 6, unit increases in household size result in increases in the probability of purchasing by 0.03% in discount stores, 0.39% in club stores, and 0.35% in dollar stores. But, in drug stores, unit increases in household size result in decreases in the probability of purchasing by 0.10%.

For household head age over 60, the probability of purchasing at dollar stores and drug stores is higher by 0.87% and 0.49% relative to the age group of 40 to 60. But the probability of purchasing at discount stores is lower by 0.07% relative to households in the age group 40 and 60.

Households who reside in urban areas have a higher probability to purchase at drug stores by 0.32%. Conversely, this probability of purchasing is lower by 0.12%, 1.11%, and 2.68% in discount stores, dollar stores and convenience stores respectively. Households who reside in rural areas have a lower probability to purchase at club stores by 2.97% and at drugstores by 0.63% than households who do not live in urban and rural areas. But households who reside in rural areas have a higher probability to purchase at discount stores by 0.10% and at dollar stores by 1.13%.

Relative to households who have graduated from college (base category), household heads who have less than a high school education have a higher probability to purchase at discount stores by 0.14% but have a lower probability to purchase at drug stores by 0.99%. Similarly, household heads who have a high school degree have a higher probability to purchase at discount stores by 0.10% but have a lower probability to purchase at drug stores by 0.32%. Households with some college education are more likely to purchase at discount stores and at dollar stores by 0.06% and 1.43% respectively.

Relative to non-Hispanic white households (base category), non-Hispanic black households are more likely to purchase at discount stores by 0.13%, at club stores by 2.08%, at dollar stores by 3.61%, at convenience stores by 2.2%, and at drug stores by 0.75%. Non-Hispanic Asian households have a higher probability to purchase at club stores by 1.36%. Non-Hispanic other households have a higher probability to purchase at dollar stores by 1.36% and at convenience stores by 3.26% stores than non-Hispanic white households. Hispanic households are more likely to purchase at discount stores by 0.10%, at dollar stores by 2.28%, at convenience stores by 1.67%, and at drug stores by 0.51%.

Unit increases in the number of club stores increase the probability of purchasing at club stores by 0.88% decrease the likelihood of purchasing at drug stores by 0.55%. Unit increase in the number of grocery stores and supercenters negatively impacts the likelihood of purchasing at discount stores by 0.01%.

Relative to households who are located in the Pacific region, households who are located in New England are more likely to purchase at convenience stores by 2.74% and at drug stores by 0.90% but are less likely to purchase at club stores by 2.35%; in the Mid-Atlantic region, are more likely to purchase at dollar stores and at convenience stores by 1.36% and 7.99% but are less likely to purchase at club stores by 2.80% relative to households located in the Pacific region; in East North Central region, have a higher probability to purchase at convenience stores by 4.00%, but lower probability to purchase at club stores by 1.27% relative to households located in the Pacific region; in the West North Central region, have higher probabilities to purchase at discount stores and convenience stores by 0.14% and 9.36% but have lower probability to purchase at club stores by 1.52%; in South Atlantic region have higher probabilities to purchase at discount stores, at dollar stores, and at conveniences stores by 0.10%, 1.35%, and 5.22% relative to households located in the Pacific region but have lower probability by 1.63% for club stores; in West South Central region have a higher probability to purchase at discount stores by 0.12% and at convenience stores by 2.61% but a lower probability to purchase at club stores by 1.37% relative to households located in the Pacific region; in the Mountain region are more likely to purchase at convenience stores by 4.95% but are less likely to purchase at drug stores by -0.57%.

## Discussion and conclusion

The purpose of this study is to examine how socio-demographic factors, spending habits, and characteristics of the retail food environment affect household expenditure across all food and beverage categories by store type. The list of socio-demographic factors includes household income; household size; age; urbanization; education; race and ethnicity; and region. Characteristics of the retail environment relate to the number of club stores, the number of convenience stores, the number of grocery stores and supercenters and the number of drug stores within the zip code area of the household. The outlets considered in this study are grocery, convenience, discount, club, drug, and dollar store types.

Prior works mainly highlighted store choice. To differentiate our study from the extant literature, we explore the factors which directly affect household food expenditure by store outlet. Indeed, Volpe, Jaenicke, and Chenarides [27] estimated the impacts of expenditure share by store format, but in our study, we quantify the magnitude of the impact of household socio-demographics, the retail food environment, and spending habits on food and beverage expenditures by diverse store types. Hence, by analyzing factors that impact household food expenditure across the aforementioned six store types, this study contributes to the economic literature. Another contribution is that our study also considers habitual persistence or spending habits, a dynamic property of household expenditure on food and beverages. However, in previous studies habitual behavior was not included in the set of explanatory variables.

To further differentiate our study from previous studies, we employ dynamic correlated random effect Tobit models to incorporate habitual purchasing behavior. The source of data for this analysis is the Nielsen Homescan Panel over the period between 2011 and 2015. Specifically, we use a balanced panel of 28,109 households who participated in the survey for all five years from 2011 to 2015. The total number of observations available for analysis is 140,545. The panel structure allows us to incorporate dynamics by including lagged dependent variables as explanatory variables to account for spending habits.

Another advantage of the use of this model is its ability to handle censoring issues. Indeed, a differentiated feature of our empirical analysis relates to transforming the dependent variables which include zero observations using the inverse hyperbolic sine method. Further, we derive the accompanying expressions for calculating conditional marginal effects and the marginal effects associated with the probability of purchasing food and beverages.

The results support the supposition of habitual spending across all store outlets. These results suggest that, within the data period 2011 to 2015, habitual spending behavior is undoubtedly a key factor in affecting nominal food and beverage expenditures across all store formats. Household income is not a statistically significant factor affecting household food and beverage expenditures in any of the respective store outlets. However, household size, age, urbanization, education, race and ethnicity, region, time-invariant socio-demographic variables, indeed are drivers of household food and beverage expenditures at the six store outlets. This finding is in line with the hypothesis of underlying household heterogeneity and in agreement with the results of Blisard, Stewart, and Jolliffe [70] and of Taylor and Villas-Boas [24].

Further, the number of convenience stores located in the zip code area of households do not significantly influence the level of food and beverage expenditures across the respective store outlets. The number of club stores located in the zip code area of households negatively impacts household expenditures made at grocery stores and drug stores. In addition, the number of grocery stores and supercenters located in the zip code area negatively impacts household food and beverage expenditures made at discount stores.

Bottom line, evidence exists to support the hypothesis that the retail environment plays a limited role in affecting household expenditures for food and beverages across store outlets. This result differs from previous findings by Kyureghian and Nayga [15] and by Taylor and Villas-Boas [24], but this result is in alignment with the work by Ver Ploeg and Wilde [71]. We acknowledge a limitation in our research pertaining to this aspect. Our retail environment variables, which represent store density in ZIP code areas and the number of specific stores by type within those areas, do not directly capture the actual distances between homes and stores. If we had access to more detailed geographical data, such as the coordinates of both homes and stores, we could explore the impact of distance from home to each store type on food expenditure and examine the relationship between distance and food expenditure by store type using multivariate methods. The inclusion of such detailed distance information would provide further insights into the spatial dynamics of consumer behavior in relation to food expenditure.

For future work, we may focus on three levels of household income, low, middle, and high to make comparisons by income level. Additional research in this area also may center attention on specific household food and beverage expenditures rather than the aggregate, for example, fresh fruits and vegetables or meat products. Particularly for low-income households, we are in position to investigate nutrition intake of households associated with the six store types by income level. As such, this research may uncover a link between store type and nutrition intake, especially useful for policies dealing with various food assistance programs. Although this research covers the period 2011 to 2015, this study establishes a baseline. Our study subsequently can be replicated using more recent data to determine the robustness of our findings. Without question, because today’s food retail environment is considerably diverse, more work is needed to understand the role of store outlets in affecting household food and beverage purchases.
